# A Speed-Accuracy Tradeoff Hierarchical Model Based on Cognitive Experiment

**DOI:** 10.3389/fpsyg.2019.02910

**Published:** 2020-01-08

**Authors:** Xiaojun Guo, Zhaosheng Luo, Xiaofeng Yu

**Affiliations:** School of Psychology, Jiangxi Normal University, Nanchang, China

**Keywords:** response time, accuracy, the speed-accuracy tradeoff, time limit, hierarchical model

## Abstract

Most tests are administered within an allocated time. Due to the time limit, examinees might have different trade-offs on different items. In educational testing, the traditional hierarchical model cannot adequately account for the tradeoffs between response time and accuracy. Because of this, some joint models were developed as an extension of the traditional hierarchical model based on covariance. However, they cannot directly reflect the dynamic relationship between response time and accuracy. In contrast, response moderation models took the residual response time as the independent variable of the response model. Nevertheless, the models enlarge the time effect. Alternatively, the speed-accuracy tradeoff (SAT) model is superior to other experimental models in the SAT experiment. Therefore, this paper incorporates the SAT model with the traditional hierarchical model to establish a SAT hierarchical model. The results demonstrated that the Bayesian Markov chain Monte Carlo (MCMC) algorithm performed well in the SAT hierarchical model of parameters by using simulation. Finally, the deviance information criterion (DIC) more preferred the SAT hierarchical model than other models in empirical data. This means that it is indispensable to add the effect of response time on accuracy, but likewise should limit the effect on the empirical data.

## Introduction

In any decision-making process, one of the most basic issues is the speed-accuracy tradeoff (SAT). In our various behaviors, the SAT is almost ubiquitous. From insects to primates, the changing trend of speed and accuracy in decision-making process is an inevitable problem. The SAT is defined as an individual’s willingness to respond slowly and makes relatively fewer errors compared to their willingness to respond quickly and makes relatively more errors. This means that low speed corresponds to higher accuracy, or high speed corresponds to lower accuracy (Heitz, 2014).

In cognitive experiments, the SAT has been studied for a long time. The relationship between response time and accuracy can be obtained by different methods. In the traditional reaction time experiment, the SAT can be obtained by six basic methods: instructions, payoffs, deadlines, time bands, response signals, and partitioning of reaction times. However, it cannot obtain complete information processing dynamics and can only provide a single time point in different experimental condition. Unlike the traditional reaction time experiment, Reed (1973) and Wickelgren (1977) proposed a SAT experimental paradigm. Compared to the traditional reaction time experiment, the SAT experiment is a different experimental paradigm. In the SAT experiment, processing time is an independent variable or an experimental condition and each experimental condition is applied to different processing times. Moreover, a speed-accuracy tradeoff model (SAT model) is used to fit the reaction time and accuracy in different experimental conditions. Therefore, SAT model can provide a complete dynamic relationship between reaction time and accuracy. After that, SAT experiment and the model were widely applied in cognitive experiments, such as conceptual processing (McElree et al., 2000), sentence comprehension (McElree, 2000; McElree et al., 2003), Memory (McElree, 1998) and Attention (McElree and Carrasco, 1999; Giordano et al., 2009).

In addition to the SAT model, the sequential sampling models are likewise used to analyze SAT experiments. In the sequential sampling models, the most popular model is the diffusion model. Furthermore, the diffusion model can interpret various SAT criterions by different parameters, such as boundary separation (Ratcliff and Rouder, 1998; Ratcliff et al., 2001; Ratcliff et al., 2003), drift rate (Starns et al., 2012; Rae et al., 2014). McElree and Dosher (1989) derived the expression for response time and accuracy from the diffusion model. In contrast with SAT model, the diffusion model was worse to fit the experimental data. In addition, the density function of the diffusion model is extremely complex (Cox and Miller, 1970), which makes more difficult to apply.

The relationship between response time and response accuracy represents an important area of study within educational testing. In educational testing, the most popular model is the hierarchical model of van der Linden (2007). Moreover, it is defined as the traditional hierarchical model in this paper. The traditional hierarchical model models the relations between speed and accuracy for a population of test takers separately from the impact of these parameters on the responses and times of the individual test takers. The same will be done for the relations between the time and response parameters of the items. Therefore, the relations between the response and time can be captured at a higher level of modeling. In other word, the traditional hierarchical model consists of two levels. The first level is two independent response models and response time model, and the second level is the joint distribution of the person parameters and the joint distribution of the item parameters. The hierarchical model links the correlation between ability and speed to account for the tradeoff between response time and accuracy. Additionally, the hierarchical model greatly promotes the analysis and application of response time and accuracy (Wang et al., 2013, 2018; Meng et al., 2014; Zhan et al., 2018). However, the traditional hierarchical model does not fully explain the relationship between the response time and accuracy. Because of this, Ranger and Ortner (2012) and Meng et al. (2015) further explained the relationship between response time and accuracy based on covariance. However, they cannot directly reflect the dynamic relationship between response time and accuracy. In contrast with covariance, a response moderation model took the residual response time as the independent variable of the response model (Bolsinova et al., 2016, 2017). Nevertheless, the response moderation model enlarged the time effect and ignored the influence of ability on accuracy.

In cognitive experiments, SAT model has obvious advantages, whereas the current hierarchical model has obvious shortcomings in the tradeoff between response time and accuracy. Therefore, a SAT hierarchical model integrates the SAT model with the traditional hierarchical model in this paper. The SAT hierarchical model not only reflects the dynamic relationship between response time and accuracy, but can also avoid the influence of expanding time on accuracy. The paper is organized as follows. Firstly, the SAT hierarchical model is described based on the SAT model. Secondly, a Bayesian estimation procedure is proposed and some simulation studies are used to evaluate parameter recovery. Thirdly, three hierarchical models are compared to an empirical data. Finally, the paper concludes with a discussion.

## SAT Hierarchical Model

In the paper, the SATHM is based on the hierarchical framework. In the SAT hierarchical model, the SAT response model is formulated by the previous response model and the SAT model. In addition, the other parts are the same with the traditional hierarchical model.

### Response Time Model

For the response times, a lognormal model is linked by the latent speed variable (τ_*i*_), the item time intensity (β_j_) and the item residual variance $\left({\text{σ}}_{\text{j}}^{2}\right)$. Within Eq. 1, lnT_*ij*_ is the response time of examinee *i* on item *j* after a log transformation.

(1) $${\mathrm{lnT}}_{\mathrm{ij}}∼N\,\left(\beta_{j}-\tau_{i},\sigma_{j}^{2}\right)$$

### SAT Response Model

In Eq. 2, SAT model is an exponential function (Reed, 1973, 1976).

(2) $$d^{\prime }\,\left(t\right)=\lambda \times \left(1-\exp\left(-\varphi \times \left(t-\delta \right)\right)\right)\;t>\delta \,\mathrm{and}\,t\neq 0$$

Where λ is the asymptotic level of accuracy, δ is the response time at which accuracy begins to grow above chance or non-decision time, φ represents the slope of the accuracy to asymptote. *d*′(*t*) is the accuracy of different response time. In each experimental condition, the three parameters of the SAT model were fitted to each observer’s response time and the average accuracy by the method of least squares. Moreover, the SAT model can determine the effect of experimental conditions by adding different parameters.

In the traditional hierarchical model, the basic assumption of the response model is that probability is not included time-limit effect. However, there is no doubt that time limits can detract from average examinee performance in that examinees correctly answered fewer items with the imposed time limits. Therefore, it is very necessary to model a response model that takes into account the impact of response time and ability. In the SAT model, λ is the asymptotic level of accuracy with no time limit. It is consistent with the assumptions of the response model in the traditional hierarchical model. Because of this, the lambda (λ) of the SAT model is defined as two-parameter logistic model (2PLM):

(3) $$\lambda_{\mathrm{ij}}=P\left(\eta_{\mathrm{ij}}=1|a_{j},b_{j};\theta_{i}\right)=\frac{\exp\left(a_{j}\times \theta_{i}-b_{j}\right)}{1+\exp\left(a_{j}\times \theta_{i}-b_{j}\right)}$$

Where η_*ij*_ is the latent response of examinee *i* for item *j*. θ_*i*_ denotes the ability parameter, *a*_*j*_ and *b*_*j*_ are the discrimination and difficulty for item *j.*

In the educational test, the tradeoffs of different test takes on the item may be different. Therefore, the parameters of the SAT model should be reconstructed. For the time term φ×(*t*−δ), it can be replaced with the term α_*j*_*Z*_*ij*_ + ζ. *Z*_*ij*_ is the standardized residual log-response time of examinee *i* for item *j*, which reflects the difference between the observation time and the expected time (Eq. 4). α_*j*_ is the slope of residual time for item *j*, and ζ is the intercept of the effect of residual time on the test. Due to the condition of *t*−δ > 0, the exponential transformation is added in the term (α_*j*_*Z*_*ij*_ + ζ). Finally, the SAT response model is established (SATM, Eq. 5). Furthermore, when the time is sufficient, the SAT response model is transformed to 2PLM. Due to response time as a random variable, the response time may be different if an examinee on the item can be answered more than once. The SATM can describe the theoretical relationship between the different response time and accuracy.

(4) $$Z_{\mathrm{ij}}=\frac{{\mathrm{lnT}}_{\mathrm{ij}}-\left(\beta_{j}-\tau_{i}\right)}{\sigma_{j}}$$

(5) $$P_{\mathrm{ij}}\left(U_{\mathrm{ij}}=1|Z_{\mathrm{ij}}\right)=\lambda_{\mathrm{ij}}(1-\exp\left(-\exp\left(\alpha_{j}Z_{\mathrm{ij}}+\zeta \right)\right)$$

**FIGURE 1:**
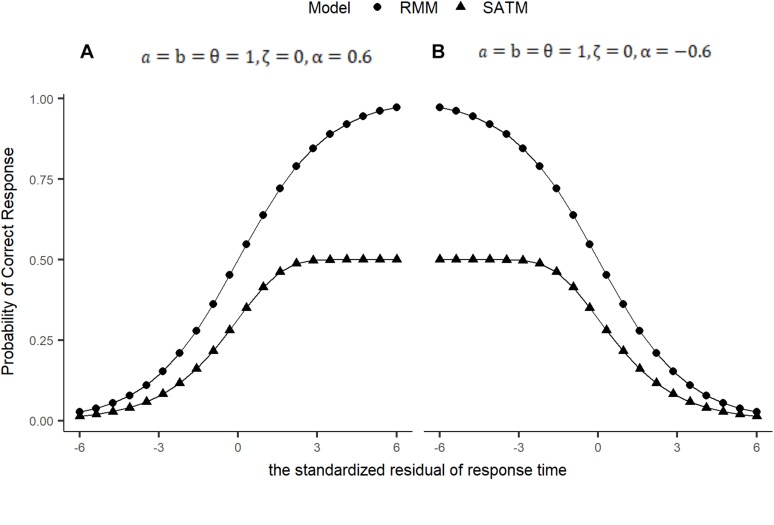
The relationship between residual time and accuracy. **(A)** α > 0, **(B)** α < 0

In order to compare the SAT response model with other models, response moderation model was Eq. 6 (RMM, Bolsinova et al., 2017). Figure 1 showed the relationship between residual time and accuracy of SATM and RMM. In figure A and B, the parameters of the two models were the same. However, there were significant differences between the two models on the asymptotic level of accuracy. The probability of RMM can always close to 1 by the increase in response time. Therefore, it means that response time has a crucial impact on accuracy. Although examinees’ ability are extremely low, they can also get a high score in the difficult item by increasing the time. In SATM, the accuracy is affected not only by response time, but also by ability. Even if the time is enough, the accuracy of SATM is also low for low-ability examinee.

(6) $$P\left(U_{\mathrm{ij}}=1|Z_{\mathrm{ij}}\right)=\frac{\exp\left(a_{j}\times \theta_{i}-b_{j}+\alpha_{j}\,Z_{\mathrm{ij}}\right)}{1+\exp\left(a_{j}\times \theta_{i}-b_{j}+\alpha_{j}\,Z_{\mathrm{ij}}\right)}$$

### Hierarchical Model Framework

The SAT hierarchical model also consists of two levels. At the first level, SATM and the response time model are two independent models. At the second level, the person parameters and item parameters are assumed to draw from a multivariate normal distribution with mean vector and covariance matrix, respectively (Eq. 7).

(7) $$\mu_{I}=\left[\mu_{\theta }\,\mu_{\tau }\right]\,\text{and}\,\Sigma_{P}=\left[\begin{array}{cc} \sigma_{\theta }^{2}\,\sigma_{\theta \,\tau } & \\ \sigma_{\theta \,\tau }\,\sigma_{\tau }^{2} & \end{array}\right]$$

(8) $$\mu_{J}=\left[\mu_{b}\,\mu_{\beta }\right]\,\text{and}\,\Sigma_{I}=\left[\begin{array}{cc} \sigma_{b}^{2}\,\sigma_{b\,\beta } & \\ \sigma_{b\,\beta }\,\sigma_{\tau }^{2} & \end{array}\right]$$

## Estimation and Model Selection

### Identifying Restrictions

To identify the SAT hierarchical model, the parameters should be fixed to μ_θ_ = μ_τ_ = 0 and $\sigma_{\theta }^{2}=\sigma_{\tau }^{2}=1$ (van der Linden, 2007).

### Prior Distributions

The SAT hierarchical model is estimated by a fully Bayesian Markov chain Monte Carlo (MCMC) method. The prior for the item parameters a_j_,1/σ_j_, and α_j_ all follow the left-truncated normal distribution *N*(0,1)I(0,). The prior for ζ is follows the standard normal distribution *N*(0,1). Moreover, the item parameters b_j_ and β_j_ are assumed to follow the normal distribution *N*(0.001,0.001). The covariance matrix Σ_I_ selects an inverse-Wishart distribution InvWishart(R_2,_ 2), where R_2_ is a binary unit matrix. Due to identifying restrictions, the correlation ρ_θτ_ is equal to the covariance σ_θτ_, and ρ_θτ_ ∈ [−1,1]. A doubly truncated normal distribution is selected as the prior distribution of the covariance σ_θτ_∼N(0,1)I(−1,1) (Meng et al., 2015).

### Model Fit for the Hierarchical Models

On the model selection criteria, the deviance information criterion (DIC, Spiegelhalter et al., 2002) is selected. Based on the posterior distribution of the log-likelihood or the deviance, DIC is calculated from the samples generated by the MCMC simulation. $\text{DIC}=\bar{D}+\text{pD}$, where pD is the effective number of parameters, $\bar{D}$ is the posterior mean of deviance (i.e., −2 × Log-likelihood). The smaller the DIC, the better the model is fitted in the empirical data.

## Simulation Study

### Design of the Simulation Study

To verify the parameter recovery with the proposed estimation method, a simulation study was carried out based on the test length (*m* = 30, 60) and the sample size (*N* = 500, 1000). There were 30 replications for each condition. For different item parameters, they were separately drawn from different distributions: a_j_∼N(0,1)I(0), $\frac{1}{{\text{σ}}_{\text{j}}}∼\text{N}\,\left(0,1\right)\,\text{I}\,\left(0\right)$, α_j_∼N(0,1)I(0), ζ∼N(0,1), and [b_j_,β_j_]∼MVN([0,3],$\left[{\text{b}}_{\text{j}},{\text{β}}_{\text{j}}\right]∼\text{MVN}\left(\left[0,3\right],\left[\begin{array}{cc} 1 & .25\\ .25 & .25 \end{array}\right]\right)$. The person parameters θ and τ were sampled from a bivariate normal distribution with σ_θτ_ = 0.5. The chosen parameters, test length and sample size are the most commonly used settings (Wang et al., 2013; Meng et al., 2015; Bolsinova et al., 2017).

### Results of the Simulation Study

The item and person parameters were measured by the Mean squared error (MSE) and average bias (Bias).

(9) $$\mathrm{MSE}\,\left(\hat{\xi }\right)=\frac{\sum_{r=1}^{R}\sum_{j=1}^{m}{\left(\hat{\xi }-\xi \right)}^{2}}{R\times m}$$

(10) $$\mathrm{Bias}\,\left(\hat{\xi }\right)=\frac{\sum_{r=1}^{R}\sum_{j=1}^{m}\left(\hat{\xi }-\xi \right)}{R\times m}$$

Where $\hat{\xi }$ and ξ are the estimated and true values of model parameters, respectively. *R* is the number of replications and *m* is the test length.

The estimated results of the item parameters are displayed in Table 1. The MSE for the item parameters decreased when the sample size *N* increased. For the condition with *N* = 1000, *m* = 60, the MSE of b decrease from 0.0592 to 0.034, and the other parameters were less than 0.032. The absolute Bias of the item parameters were close to 0.07. Therefore, the results of item parameters were acceptable for all conditions.

**TABLE 1:** MSE and Bias for the item parameters.

		***N* = 500, *m* = 30**		***N* = 1000, *m* = 30**		***N* = 500, *m* = 60**		***N* = 1000, *m* = 60**	
					
**Model parameters**		**MSE**	**Bias**	**MSE**	**Bias**	**MSE**	**Bias**	**MSE**	**Bias**
Item parameters	*a*	0.0592	–0.0279	0.0315	–0.0338	0.0460	–0.0334	0.0314	–0.0350
	b	0.0592	0.0423	0.0295	0.0115	0.0564	–0.0003	0.0340	–0.0046
	ζ	0.0032	0.0186	0.0092	0.0611	0.0110	–0.0100	0.0004	0.0047
	α	0.0551	0.0102	0.0663	0.0019	0.0590	0.0085	0.0256	0.0148
	σ	0.0018	–0.0007	0.0008	0.0018	0.0016	–0.0007	0.0009	0
	β	0.0048	–0.0068	0.0019	0.0146	0.0068	–0.0116	0.0025	–0.0087
Person parameters	θ	0.1710	0.0174	0.1770	–0.0019	0.0918	0.0031	0.0948	–0.0031
	τ	0.0298	–0.0002	0.0258	0.0050	0.0151	0.0053	0.0147	0.0142

Alternatively, Table 1 shows the result of the person parameters. The MSE of the speed parameter was below 0.03 within each condition. However, the result of the ability decreased from 0.17 to less than 0.10 with the increase of the test length. On the other hand, the Bias of the person parameters fluctuated around zero. Consequently, the person parameters were likewise acceptable.

## Empirical Example

### Data and Method

We analyzed data from the Raven’s Standard Progressive Matrices (SPM). The SPM includes five sets (A to E) and 12 items in each set. The valid sample size was 320 and the difficulty of the items was disorderly. In the process of responding, examinees could only answer questions in the order of the presented, and were not allowed to be returned. The time limit of this test was 40 min.

Three models were fitted to the empirical data using Gibbs samplers (30000 iterations, 10000 burn-in, 2 chains and 2 thinning). The multivariate potential scale reduction factor (Brooks and Gelman, 1998) was used to monitor the convergence diagnostic and required less than 1.1.

## Results

The SPM data was fitted by the traditional hierarchical model (van der Linden, 2007, M0), the RMM hierarchical model (RMHM) and the speed-accuracy tradeoff hierarchical model (SATHM), respectively. According to the DIC, SATHM was the smallest (DIC = 47306.22), RMHM was followed (DIC = 47677.85) and the largest was M0 (DIC = 48069.24). Therefore, it means that considering the effect of response time on accuracy can improve model fit. Furthermore, SATHM fitting is superior to RMHM, so it is necessary to limit the effect of response time on accuracy. The remainder of this section will focus on the results of SATHM.

The results of the hyperparameters and the intercept parameter (ζ) are presented in Table 2. With the 95% credible interval for the correlation σ_*θτ*_, speed was negatively correlated with ability. The mean of the intercept parameter (ζ) was 2.6431 and the mean of b was −2.646. Meanwhile, the correlation of item parameters b and β was highly positive.

**TABLE 2:** Posterior means and 95% credible intervals of the hyperparameters and the intercept parameter (ζ) under the SAT hierarchical model.

	**Mean**	**s.d**	**95% credible interval**
σ_θτ_	–0.7361	0.0544	[−0.8239 −0.6109]
${\text{σ}}_{\text{b}}^{2}$	2.2172	0.4404	[1.5157 3.2314]
σ_bβ_	0.8467	0.1818	[0.5612 1.2672]
${\text{σ}}_{\text{>β}}^{2}$	0.4849	0.0916	[0.3367 0.6966]
ζ	2.6431	0.1385	[2.3960 2.9316]
μ_b_	–2.6460	0.2019	[−3.0521 −2.2519]
μ_β_	2.4868	0.0983	[2.2950 2.6783]

**FIGURE 2:**
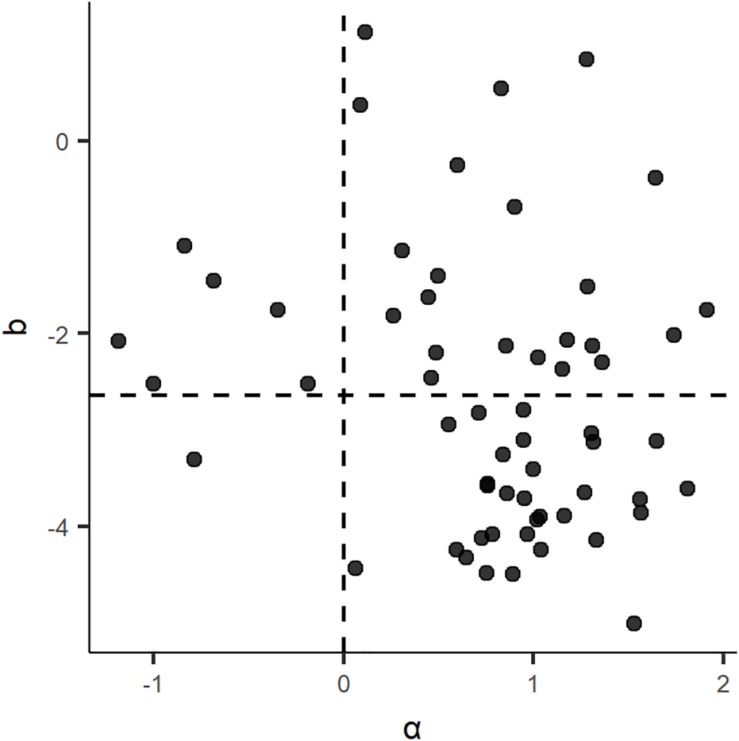
Posterior means of the b and α under SATHM.

Finally, the relationship between b and alpha is presented in Figure 2. The dotted line of the horizontal axis is the mean of b. From Figure 2, when α was less than 0, b was greater than or approaching the mean of b for all items. Therefore, the effect of residual response time is more likely to be negative for medium-difficulty items. The result is slightly different from that of Bolsinova et al. (2017; Figure 3). It may be related to the difficulty of the test, because the test is relatively simple.

## Discussion

The accuracy of completing a task has always been the main evaluation index in the educational assessment. During a variety of task situations, all the indexes indicating the quality of examinees are extremely important, including the correctness of the result as well as the timeliness of the decision-making process. Moreover, most tests are administered within an allocated time. Due to the time limit, examinees might have different tradeoffs on different items. However, current models cannot effectively analyze the effect of the SAT. In cognitive experiments, SAT model is more superior to describing the dynamic relationship between reaction time and accuracy than other models. Therefore, this paper incorporates the SAT model with the traditional hierarchical model to establish the SATHM. In addition, the parameters of SATHM can be performed well using the MCMC algorithm and the DIC more preferred the SATHM than other models in empirical data.

Some other issues should be further researched. Firstly, the SATHM merely explains the item-specific tradeoff. However, it is simple to extend to the tradeoff of between-person differences with reference to Bolsinova et al. (2016, 2017). Secondly, the lognormal response model was selected to model the response time in SATHM, but it not always satisfies the normality assumption. Therefore, some other models should be investigated, such as Shifted Wald distribution (Anders et al., 2016) and the semi-parameter model (Wang et al., 2013). Finally, Chen et al. (2018) have explored the relationships between response time and accuracy and found that there may be a curvilinear dependency. Accordingly, a curvilinear SATHM can be obtained with some extensions.

## Data Availability Statement

All datasets generated for this study are included in the article/Supplementary Material.

## Author Contributions

XG: design the study, data analysis, manuscript writing, and revision. ZL: preliminary idea construction, manuscript revision, and proofreading. XY: manuscript revision, and proofreading.

## Conflict of Interest

The authors declare that the research was conducted in the absence of any commercial or financial relationships that could be construed as a potential conflict of interest.
