# Supplementary material for: A Speed-Accuracy Tradeoff Hierarchical Model Based on Cognitive Experiment
Source: Front Psychol. 2020 Jan 8;10:2910. doi: 10.3389/fpsyg.2019.02910 (PMC6960267; doi:10.3389/fpsyg.2019.02910)
Supplement: Supplementary file 1 [file Table_1.docx]

Supplementary Material(jags syntax)

model{

for(i in 1:N){

for(j in 1:m){

eta[i,j]<-exp(a[j]*thtao[i,1]-bbeta[j,1])

pA[i,j]<-eta[i,j]/(1+eta[i,j]) # the probability of latent response

t0[i,j]<-(bbeta[j,2]-thtao[i,2])

RT[i,j]~dnorm(t0[i,j],sigT[j]) #Response Time model

z[i,j]<-(RT[i,j]-t0[i,j])*siga[j]

pT[i,j]<-(1-exp(-exp(alpha[j]*z[i,j]+d)))# the influence of time on probability

p[i,j]<-pA[i,j]*pT[i,j] # SATM

U[i,j]~dbern(p[i,j]) # Observation response

}

thtao[i,1:2]~dmnorm(muth[1:2],thcov[1:2,1:2])

}

for(j in 1:m){

bbeta[j,1:2]~dmnorm(mubb[1:2],bbcov[1:2,1:2])

a[j]~dnorm(0,1)T(0,)

siga[j]~dnorm(0,1)T(0,)

sigT[j]<-pow(siga[j],2)

alpha[j]~dnorm(0,1)

}

muth[1]<-0

muth[2]<-0

Rth[1,1]<-1

Rth[2,2]<-1

Rth[1,2]~dnorm(0,1)T(-1,1)

Rth[2,1]<-Rth[1,2]

mubb[1]~dnorm(0.01,0.01)

mubb[2]~dnorm(0.01,0.01)

Rbb[1,1]<-1

Rbb[2,2]<-1

Rbb[1,2]<-0

Rbb[2,1]<-0

d~dnorm(0,1)T(0,)

bbcov[1:2,1:2]~dwish(Rbb[1:2,1:2],2)

sigbb[1:2,1:2]<-inverse(bbcov[1:2,1:2])

thcov[1:2,1:2]<-inverse(Rth[1:2,1:2])

}
